# Prevalence and risk factors for postnatal mental health problems in mothers of infants admitted to neonatal care: analysis of two population-based surveys in England

**DOI:** 10.1186/s12884-023-05684-5

**Published:** 2023-05-22

**Authors:** Jenny Gong, Gracia Fellmeth, Maria A. Quigley, Chris Gale, Alan Stein, Fiona Alderdice, Siân Harrison

**Affiliations:** 1 NIHR Policy Research Unit in Maternal and Neonatal Health and Care, National Perinatal Epidemiology Unit, Nuffield Department of Population Health, University of Oxford, Old Road Campus, Headington, Oxford, UK; 2 Neonatal Medicine, School of Public Health, Faculty of Medicine, Imperial College London, Chelsea and Westminster Campus, 369 Fulham Road, London, UK; 3 Department of Psychiatry, Medical Sciences Division, University of Oxford, Oxford, UK; 4 Medical Research Council/Wits University Rural Public Health and Health Transitions Research Unit (Agincourt), School of Public Health, Faculty of Health Sciences, University of the Witwatersrand, Johannesburg, South Africa; 5 African Health Research Institute, Durban, KwaZulu-Natal South Africa; 6 School of Nursing and Midwifery, Queens University Belfast, Belfast, UK

**Keywords:** Neonatal unit, Perinatal, Depression, Anxiety, Posttraumatic stress, Maternity survey, Mental health

## Abstract

**Background:**

Previous research suggests that mothers whose infants are admitted to neonatal units (NNU) experience higher rates of mental health problems compared to the general perinatal population. This study examined the prevalence and factors associated with postnatal depression, anxiety, post-traumatic stress (PTS), and comorbidity of these mental health problems for mothers of infants admitted to NNU, six months after childbirth.

**Methods:**

This was a secondary analysis of two cross-sectional, population-based National Maternity Surveys in England in 2018 and 2020. Postnatal depression, anxiety, and PTS were assessed using standardised measures. Associations between sociodemographic, pregnancy- and birth-related factors and postnatal depression, anxiety, PTS, and comorbidity of these mental health problems were explored using modified Poisson regression and multinomial logistic regression.

**Results:**

Eight thousand five hundred thirty-nine women were included in the analysis, of whom 935 were mothers of infants admitted to NNU. Prevalence of postnatal mental health problems among mothers of infants admitted to NNU was 23.7% (95%CI: 20.6–27.2) for depression, 16.0% (95%CI: 13.4–19.0) for anxiety, 14.6% (95%CI: 12.2–17.5) for PTS, 8.2% (95%CI: 6.5–10.3) for two comorbid mental health problems, and 7.5% (95%CI: 5.7–10.0) for three comorbid mental health problems six months after giving birth. These rates were consistently higher compared to mothers whose infants were not admitted to NNU (19.3% (95%CI: 18.3–20.4) for depression, 14.0% (95%CI: 13.1–15.0) for anxiety, 10.3% (95%CI: 9.5–11.1) for PTS, 8.5% (95%CI: 7.8–9.3) for two comorbid mental health problems, and 4.2% (95%CI: 3.6–4.8) for three comorbid mental health problems six months after giving birth. Among mothers of infants admitted to NNU (*N* = 935), the strongest risk factors for mental health problems were having a long-term mental health problem and antenatal anxiety, while social support and satisfaction with birth were protective.

**Conclusions:**

Prevalence of postnatal mental health problems was higher in mothers of infants admitted to NNU, compared to mothers of infants not admitted to NNU six months after giving birth. Experiencing previous mental health problems increased the risk of postnatal depression, anxiety, and PTS whereas social support and satisfaction with birth were protective. The findings highlight the importance of routine and repeated mental health assessments and ongoing support for mothers of infants admitted to NNU.

**Supplementary Information:**

The online version contains supplementary material available at 10.1186/s12884-023-05684-5.

## Introduction

Each year, approximately 1 in 7 infants born in the United Kingdom (UK) is admitted to a neonatal unit (NNU), which provides integrated services to sick and preterm infants who are in need of specialist medical care [1]. In addition to fear and anxiety surrounding the health of their infant, the NNU can also be a stressful environment for parents [2]. The physical appearance of the ill infant, exposure to intrusive medical equipment, along with potential miscommunications with staff can all contribute to parental distress [3]. Notably, some mothers of NNU infants report feelings of hopelessness and a sense of “loss of the maternal role,” along with doubts regarding their ability to care for their baby [3]. The confluence of these environmental and psychological factors may increase the risk of mental health problems among parents of infants admitted to NNU [2]. Perinatal mental health problems affect not only the mother but may also negatively impact infants’ health and development across the life course [4]. Previous research suggests that maternal mental health problems are associated with a decrease in the quality of parent–child relationships, poor infant outcomes such as impairments in neurocognitive and motor development, along with delays in language development [4].

According to results from a systematic review conducted by Vigod et al., [5] prevalence of postnatal depression among mothers of infants admitted to NNU ranges from 28 to 70%, depending on the diagnostic criteria used. In another systematic review by Malouf et al., [2] prevalence of postnatal anxiety among parents whose infants were admitted to NNU was as high as 42% one month after birth, and prevalence of postnatal post-traumatic stress (PTS) one month after birth was similar at 40%. Although these rates gradually decreased over time, both anxiety and PTS rates remained more prevalent and persistent in parents of infants admitted to NNU, as compared to figures reported for women in the general perinatal population [2]. Past research has estimated that mothers of infants admitted to NNU experience approximately 2–3 times higher prevalence of postnatal depression, anxiety, and PTS compared to the general perinatal population [6, 7]. However, high heterogeneity and small sample sizes across studies examining mental health problems in mothers of NNU infants limit the reliability of prevalence estimates [2].

In addition to the limitations of existing evidence, few studies have reported prevalence of comorbidity of perinatal mental health problems among parents of infants admitted to NNU. Mental health problems commonly co-occur; a meta-analysis conducted by Falah-Hassani et al. [8] estimated that 13.1% of women in the general perinatal population experience comorbid depressive and anxiety symptoms within 8 weeks after childbirth. One small study conducted in the USA by Das et al. [9] reported comorbid postnatal depression, anxiety, and stress in 15.6% of a sample of mothers of infants admitted to NNU. However, the overall evidence for the prevalence of comorbid mental health problems in mothers of NNU infants is sparse.

Factors such as history of mental health problems, lack of social support, unplanned pregnancy, stressful life events, and interpersonal violence have been consistently linked with increased postnatal anxiety and depression [7] in the general perinatal population. Recent immigration, childcare stress, along with low partner support have also been linked to an increased risk of comorbidity of mental health problems [8]. Additionally, results from a meta-analysis by Grekin and O’Hara demonstrate that major depression and traumatic childbirth experiences are associated with increased risk of PTS among postnatal mothers [10]. However, it is unknown whether these and/or other unique factors are associated with mental health problems in mothers of infants admitted to NNU, as there is limited research exploring risk factors for postnatal depression, anxiety, PTS, and comorbidity of these mental health problems in this specific population [6].

Therefore, the aims of this study are to: 1) provide up-to-date prevalence estimates of postnatal depression, anxiety, PTS, and comorbidity of these mental health problems in mothers of infants admitted to NNU, compared to the prevalence rates among mothers whose infants were not admitted to NNU; 2) explore sociodemographic, pregnancy- and birth-related factors associated with postnatal depression, anxiety, PTS, and comorbidity of these mental health problems for mothers of infants admitted to NNU. Such data are critical in guiding future research, informing clinical guidelines as well as policy decisions.

## Methods

### Study design and sample

This study was a secondary analysis of data from the 2018 and 2020 National Maternity Surveys (NMS), which were cross-sectional, population-based postal surveys carried out by the National Perinatal Epidemiology Unit (NPEU) in England [11, 12]. The NMS aim to assess women’s experiences of maternity care around the time of pregnancy and birth, where participants have the choice to complete the survey: 1) on paper; 2) online; or 3) by telephone with an interpreter. Details about the larger study have been described elsewhere [11, 12]. Briefly, a sample of 16,000 women in 2018 were randomly sampled using birth registration records by the Office for National Statistics (ONS), while 16,050 women were sampled in 2020 [11, 12]. All women in the sample lived in England at the time of survey completion, were aged 16 and over, and had given birth during a two-week interval in October 2017 (for the 2018 survey) or May 2020 (for the 2020 survey) [11, 12]. These specific time intervals were selected to identify women who had given birth six months prior to survey administration [11, 12]. Women whose baby had died were not included in the study [11, 12]. NNU admission (yes, no) was assessed through a direct question within the NMS.

### Postnatal depression, anxiety, and PTS

Postnatal depression, anxiety, and PTS were assessed within the NMS using standardised self-report measures. Postnatal depression was assessed using the Edinburgh Postnatal Depression Scale (EPDS). The EPDS consists of 10 items each scored from 0–3, with a total score of 30, where a higher number indicates greater severity of symptoms [13]. To identify individuals with ‘probable depression,’ a cut-off value of ≥ 13 was used, with a sensitivity of 79% and specificity of 89% [13]. Women scoring above the threshold were categorized as having probable depression.

Postnatal anxiety was assessed using the two-item Generalized Anxiety Disorder scale (GAD-2), which asks respondents to recall the frequency with which they have experienced the following anxiety symptoms over the past two weeks: 1) “feeling nervous, anxious, or on edge;” 2) “not being able to stop or control worrying.” Items are scored from 0 to 3 (0 = not at all, 3 = nearly every day) [14]. A total score of ≥ 3 is used to classify women with clinically significant symptoms of anxiety, which corresponds with 86% sensitivity and 83% specificity [14].

The Primary Care Posttraumatic Stress Disorder Screen for DSM-IV (PC-PTSD-IV) was adapted and integrated into the 2018 NMS to identify women with PTS [15]. The tool encompasses four items: 1) re-experiencing traumatic events; 2) emotional numbing; 3) avoidance; and 4) hyperarousal. The Primary Care PTSD Screen for DSM-5 (PC-PTSD-5) was used in 2020 as this tool reflects more recent DSM-5 criteria [16]. Compared to the PC-PTSD-IV, the PC-PTSD-5 includes an additional item on feelings of guilt for causing the event(s) to happen [16]. All items are scored dichotomously (0 = no, 1 = yes) with a pre-determined threshold of ≥ 3, which corresponds with a sensitivity and specificity of 78% for the PC-PTSD-IV, compared to a sensitivity of 93% and specificity of 85% for the PC-PTSD-5 [15, 16]. Women who scored above this threshold were categorized as experiencing PTS.

Within this study, comorbidity of mental health problems is defined as individuals who scored above the thresholds, as detailed above, for two or three mental health problems (postnatal anxiety, depression, and/or PTS).

### Risk factors

Potential risk factors for postnatal mental health problems in mothers of infants admitted to NNU were identified from the literature and those for which data were available from the NMS were included in this study. The sociodemographic factors included were: age group (≤ 24 years, 25–29 years, 30–34 years, 35 + years); country of birth (UK, outside UK); ethnicity (White-British, White-Other, Mixed, Asian, Chinese, Black, Other); level of area deprivation measured by the index of multiple deprivation (IMD) grouped into quintiles (1 [least socio-economically advantaged] to 5 [most socio-economically advantaged]); age when leaving full-time education (≤ 16 years, 17–18 years, ≥ 19 years); living with partner (yes, no); long-term mental health problem (yes, no); and social support (measured on a Likert scale from 0 [not at all supported] to 6 [completely supported]). The pregnancy- and birth-related factors included were: parity (primiparous, multiparous); multiplicity (singleton, multiple birth); pregnancy planning (planned, unplanned); satisfaction with labour and birth (scores of 0 [least satisfied] to12 [most satisfied] on the 6-item birth satisfaction scale-revised indicator (BSS-RI) (2020 survey) or on 6 of the 10 items on the BSS-R (2018 survey) [17]); gestational age (very pre-term [< 32 weeks], pre-term [32–36 weeks], term [37 + weeks]); birthweight (very low [< 1500 g], low [1500-2500 g], normal [2500 g +]); length of NNU stay (24 h or less, 25 h to 7 days; 8 to 28 days; more than 28 days); mode of birth (vaginal, instrumental, planned caesarean, unplanned caesarean); self-reported antenatal anxiety (yes, no); self-reported antenatal depression (yes, no); smoking during pregnancy (yes, no); household or passive smoking during pregnancy (yes, no). Ethnicity was collapsed into “White-British” or “other” for the regression analyses. Further details on the assessment of these factors are available in the published NMS reports [10, 11].

### Statistical analysis

Datasets from 2018 and 2020 were cleaned prior to merging. Descriptive statistics were used to describe the characteristics of survey participants and to estimate the prevalence of postnatal depression, anxiety, and PTS for mothers whose infants were admitted to NNU (*N* = 935), with 95% confidence intervals (CI). The proportion of mothers whose infants were admitted to NNU having one postnatal mental health problem (depression, anxiety, or PTS), two postnatal mental health problems (any two of depression, anxiety, or PTS), and three postnatal mental health problems (depression, anxiety, and PTS) was also estimated. For comparison, corresponding prevalence estimates were calculated for mothers whose infants were not admitted to NNU (*N* = 7,604).

While prevalence estimates included a comparison between mothers of infants admitted to NNU and mothers of infants not admitted to NNU, analysis of risk factors was restricted to mothers of NNU infants. As study outcomes were not rare (prevalence > 10%), analyses were conducted using modified Poisson regression to estimate risk ratios (RR) [18]. Modified Poisson regression was used to estimate the unadjusted associations between risk factors and each mental health outcome separately (postnatal depression, anxiety, and PTS) among mothers of infants admitted to NNU. For each outcome, factors significant at the univariable stage (*p* < 0.10) were subsequently fitted in multivariable models. Multivariable model building was conducted in three stages. First, multivariable models were adjusted for sociodemographic risk factors only. Next, models were adjusted for pregnancy- and birth-related risk factors only. Finally, both models were combined, removing factors that were not statistically significant (*p* > 0.05). Therefore, only risk factors significant (*p* < 0.05) after mutually adjusting for all other variables were retained in the final models. All models were adjusted for survey year.

Multinomial logistic regression was used to investigate which sociodemographic and pregnancy- and birth-related risk factors were associated with having one postnatal mental health problem or two or three postnatal mental health problems among mothers of infants admitted to NNU, using unadjusted and adjusted odds ratios (OR). The same univariable and multivariable model building process described above was repeated for multinomial logistic regression.

All analyses were conducted in Stata version 17 and survey weighted commands were used to reduce the effect of non-response bias [11, 12]. Less than 5% of data were missing for the majority of variables [11, 12], thus complete case analysis was performed.

## Results

### Characteristics of survey participants

Questionnaires were returned by 9,120 women (4,509 in 2018 and 4,611 in 2020), corresponding to response rates of 29.0% in 2018 and 28.9% in 2020 [16, 17]. Complete data on NNU admission and postnatal depression, anxiety, and PTS were available for 8,539 women. Table 1 presents the baseline characteristics of these women overall and according to whether they did (*N* = 935) or did not (*N* = 7,604) have infants admitted to NNU. The majority of respondents were between 30 and 34 years of age (34.2%) and born within the UK (72.3%). Approximately three quarters of respondents self-identified as White-British (70.3%) and 58.5% were at least 19 years of age when they left full-time education. Most respondents reported living with their partner at the time of survey administration (82.6%) and 33.9% lived in the most socio-economically advantaged areas (top two quintiles of IMD). Around half of respondents were first-time mothers (45.9%) and the majority reported planned pregnancies (74.6%) along with singleton births (98.2%). More than half of respondents had a vaginal delivery (59.3%), and most reported full-term (93.3%) infants with normal birthweight (93.5%).

**Table 1:** Baseline characteristics of study participants in 2018 and 2020, overall and by infant NNU admission

**Baseline characteristics**	**NNU admission (*N* = 935)** n (%)	**No NNU admission (*N* = 7604)** n (%)	**Total** **(*N* = 8539)** n (%)
***Age group***			
< *24 years*	93 (17.7%)	606 (14.2%)	699 (14.5%)
*25–29 years*	213 (25.3%)	1709 (25.5%)	1922 (25.5%)
*30–34 years*	337 (31.5%)	2953 (34.5%)	3290 (34.2%)
*35* + *years*	292 (25.5%)	2336 (25.8%)	2628 (25.8%)
*Missing*	0 (0.0%)	0 (0.0%)	0 (0.0%)
***Country of birth***			
*UK*	734 (69.0%)	6049 (72.7%)	6783 (72.3%)
*Outside UK*	197 (31.0%)	1520 (27.3%)	1717 (27.7%)
*Missing*	4 (0.4%)	35 (0.5%)	39 (0.5%)
***Ethnicity***			
*White-British*	697 (68.7%)	5744 (70.5%)	6441 (70.3%)
*White-Other*	83 (11.8%)	776 (12.5%)	859 (12.4%)
*Mixed*	16 (2.0%)	173 (2.7%)	189 (2.6%)
*Asian*	68 (9.3%)	486 (8.2%)	554 (8.3%)
*Chinese*	16 (1.8%)	126 (2.2%)	142 (2.2%)
*Black*	28 (6.3%)	159 (3.9%)	187 (4.1%)
*Missing*	27 (2.9%)	140 (1.8%)	167 (2.0%)
***IMD quintile***			
* (Least advantaged) 1*	164 (29.2%)	1110 (24.9%)	1274 (25.4%)
*2*	192 (23.1%)	1427 (21.7%)	1619 (21.9%)
*3*	187 (18.0%)	1587 (19.0%)	1774 (18.9%)
*4*	209 (16.6%)	1771 (18.3%)	1980 (18.1%)
* (Most advantaged) 5*	183 (13.2%)	1709 (16.1%)	1892 (15.8%)
*Missing*	0 (0.0%)	0 (0.0%)	0 (0.0%)
***Age when leaving education***			
*16 years or less*	111 (14.7%)	823 (14.1%)	934 (14.2%)
*17–18 years*	247 (27.2%)	1894 (27.4%)	2141 (27.4%)
*19 years or over*	564 (58.1%)	4836 (58.5%)	5400 (58.5%)
*Missing*	13 (1.4%)	51 (0.7%)	64 (0.8%)
***Living with partner***			
*Yes*	836 (81.1%)	6870 (82.8%)	7706 (82.6%)
*No*	99 (19.0%)	734 (17.2%)	833 (17.4%)
*Missing*	0 (0.0%)	0 (0.0%)	0 (0.0%)
***Parity***			
*Primiparous*	595 (58.0%)	3802 (44.3%)	4397 (45.9%)
*Multiparous*	324 (42.0%)	3657 (55.7%)	3981 (54.1%)
*Missing*	16 (1.7%)	145 (1.9%)	161 (1.9%)
***Multiplicity***			
*Singleton*	877 (94.6%)	7482 (98.7%)	8359 (98.2%)
*Multiple birth*	57 (5.4%)	105 (1.3%)	162 (1.8%)
*Missing*	1 (0.1%)	17 (0.2%)	18 (0.2%)
***Pregnancy Planning***			
*Planned pregnancy*	735 (73.0%)	6137 (74.8%)	6872 (74.6%)
*Unplanned pregnancy*	189 (27.0%)	1403 (25.2%)	1592 (25.4%)
*Missing*	11 (1.2%)	64 (0.8%)	75 (0.9%)
***Gestation at birth***			
*Very pre-term*	62 (6.1%)	0 (0.0%)	62 (0.7%)
*Pre-term*	233 (25.5%)	242 (3.6%)	475 (6.0%)
*Term*	630 (68.4%)	7273 (96.4%)	7903 (93.3%)
*Missing*	10 (1.1%)	89 (1.2%)	99 (1.2%)
***Birthweight***			
*Very low birthweight*	56 (6.1%)	0 (0.0%)	56 (0.7%)
*Low birthweight*	207 (22.9%)	256 (3.7%)	463 (5.8%)
*Normal birthweight*	647 (71.0%)	7143 (96.3%)	7790 (93.5%)
*Missing*	25 (2.7%)	205 (2.7%)	230 (2.7%)
***Mode of birth***			
*Vaginal*	365 (42.2%)	4372 (61.4%)	4737 (59.3%)
*Instrumental*	143 (14.0%)	1082 (12.2%)	1225 (12.4%)
*Planned caesarean*	154 (16.7%)	1142 (14.4%)	1296 (14.7%)
*Unplanned caesarean*	268 (27.1%)	965 (12.0%)	1233 (13.7%)
*Missing*	5 (0.5%)	43 (0.6%)	48 (0.6%)

n (unweighted)

% (weighted)

Although sociodemographic characteristics were similar for mothers of infants admitted to NNU and mothers of infants not admitted at baseline, the samples differed on several pregnancy- and birth-related factors. Unsurprisingly, compared to mothers of non-NNU infants, mothers of infants admitted to NNU were more likely to be first-time mothers (58.0%) and to have had an unplanned caesarean (27.1%). Infants admitted to NNU were also more likely to be very preterm (6.1%) or preterm (25.5%) and to have low (22.9%) or very low birthweight (6.1%), compared to infants not admitted to NNU.

### Prevalence of postnatal depression, anxiety, PTS, and comorbidities

Overall, 31.1% (95% CI: 27.6, 34.7) of mothers whose infants were admitted to NNU reported one or more postnatal mental health problems. Table 2 summarises the prevalence of postnatal depression, anxiety, and PTS for respondents six months after giving birth, and provides a comparison between mothers whose infants were admitted to NNU and mothers whose infants were not admitted. The overall prevalence of depression six months after giving birth was 23.7% (95% CI: 20.6, 27.2) for mothers of infants admitted to NNU, compared to 19.3% (95% CI: 18.3, 20.4) for mothers whose infants were not admitted to NNU. Prevalence of anxiety and PTS six months after giving birth was 16.0% (95% CI: 13.4, 19.0) and 14.6% (95% CI: 12.2, 17.5) respectively for mothers of infants admitted to NNU, compared to 14.0% (95% CI: 13.1, 15.0) and 10.3% (95% CI: 9.5, 11.1) for mothers whose infants were not admitted to NNU. In total, 15.3% (95% CI: 12.6, 18.4) of mothers whose infants were admitted to NNU reported one postnatal mental health problem, 8.2% (95% CI: 6.5, 10.3) reported two postnatal mental health problems, and 7.5% (95% CI: 5.7, 10.0) reported three postnatal mental health problems six months after giving birth. In comparison, 14.0% (95% CI: 13.1, 15.0), 8.5% (95% CI: 7.8, 9.3) and 4.2% (95% CI: 3.6, 4.8) of mothers whose infants were not admitted to NNU reported one, two, or three postnatal mental health problems, respectively.

**Table 2:** Postnatal mental health problems among mothers whose infants were and were not admitted to NNU

Postnatal mental health problem	NNU Admission			No NNU Admission		
	N	%	95% CI	N	%	95% CI
***Postnatal depression***	211	23.7%	(20.6–27.2)	1339	19.3%	(18.3–20.4)
***Postnatal anxiety***	136	16.0%	(13.4–19.0)	981	14.0%	(13.1–15.0)
***Postnatal PTS***	134	14.6%	(12.2–17.5)	689	10.3%	(9.5–11.1)
***One mental health problem***	142	15.3%	(12.6–18.4)	1024	14.0%	(13.1–15.0)
***Two mental health problems***	81	8.2%	(6.5–10.3)	610	8.5%	(7.8–9.3)
***Three mental health problems***	59	7.5%	(5.7–10.0)	255	4.2%	(3.6–4.8)
***One or more mental health problems***	282	31.1%	(27.6–34.7)	1889	26.7%	(25.5–27.9)

### Factors associated with postnatal depression six months after giving birth for mothers whose infants were admitted to NNU

Regression analyses were carried out for postnatal depression, anxiety, and PTS separately. Table 3 shows the prevalence of postnatal depression for mothers whose infants were admitted to NNU, according to sociodemographic and pregnancy- and birth-related factors, along with unadjusted and adjusted RRs. At the univariable stage, factors significantly associated with postnatal depression were social support, having a long-term mental health problem, satisfaction with labour and birth, length of NNU stay, antenatal anxiety, antenatal depression, and smoking during pregnancy. In the multivariable model, the risk factors which remained significantly associated with postnatal depression after adjusting for all other factors were antenatal anxiety (aRR 1.65; 95% CI 1.19, 2.27) and having a long-term mental health problem (aRR 2.15; 95% CI 1.59, 2.90). Having social support (aRR 0.78; 95% CI 0.73, 0.84) and higher satisfaction with labour and birth (aRR 0.94; 95% CI 0.90, 0.97) emerged as protective factors against postnatal depression six months after birth.

**Table 3:** Risk ratios showing association between postnatal depression and sociodemographic and pregnancy and birth-related factors

	**Total** ***N* = 935**	**Postnatal Depression** **n (%)**		**uRR (95% CI)**	aRR (95% CI)**
**Sociodemographic factors**					
***Age group***					
≤ *24 years*	93	29 (30.1%)		1.23 (0.80–1.89)	NS
*25–29 years*	213	45 (21.8%)		0.89 (0.62–1.29)	
*30–34 years*	337	77 (24.4%)		1	
*35* + *years*	292	60 (20.5%)		0.84 (0.61–1.17)	
*** Country of birth***					
*UK*	734	172 (24.6%)		1	NS
*Outside UK*	197	37 (21.7%)		0.88 (0.61–1.28)	
*** Ethnicity***					
*White-British*	780	177 (24.1%)		1	NS
*Other*	128	27 (21.5%)		0.89 (0.59–1.34)	
*** IMD quintile***					
*(Least advantaged) 1*	164	44 (25.3%)		1.25 (0.82–1.90)	NS
*2*	192	43 (24.3%)		1.20 (0.76–1.89)	
*3*	187	43 (22.6%)		1.12 (0.73–1.70)	
*4*	209	45 (24.3%)		1.20 (0.80–1.80)	
*(Most advantaged) 5*	183	36 (20.2%)		1	
*** Age when leaving education***					
*16 years or less*	111	37 (31.2%)		1.39 (0.97–2.00)	NS
*17–18 years*	247	56 (23.3%)		1.04 (0.75–1.45)	
*19 years or over*	564	115 (22.4%)		1	
*** Living with partner***					
*Yes*	836	186 (23.9%)		1	NS
*No*	99	25 (23.2%)		0.97 (0.61–1.55)	
*** Social support ***^***#***^					
	932 (6, 4–7)	209 (4,3–6)		0.76 (0.72–0.81)*	0.78 (0.73–0.84)
*** Long-term mental health problem***					
*Yes*	118	70 (60.8%)		3.38 (2.64–4.33)*	2.15 (1.59–2.90)
*No*	810	139 (18.0%)		1	1
**Pregnancy and birth related factors**					
***Parity***					
*Primiparous*	595	125 (22.9%)	1		NS
*Multiparous*	324	83 (25.3%)	1.11 (0.83–1.47)		
***Multiplicity***					
*Singleton*	877	200 (24.0%)	1		NS
*Multiple birth*	57	11 (19.0%)	0.79 (0.44–1.43)		
***Pregnancy Planning***					
*Planned*	735	154 (22.5%)	1		NS
*Unplanned*	189	56 (28.1%)	1.25 (0.92–1.70)		
***Satisfaction with labour and birth ***^***#***^					
	888 (7, 5–9)	200 (6, 4–8)	0.89 (0.85–0.92)*		0.94 (0.90–0.97)
***Gestation at birth***					
*Very pre-term*	62	14 (28.6%)	1.21 (0.75–1.96)		NS
*Pre-term*	233	50 (23.6%)	1.00 (0.70–1.43)		
*Term*	630	144 (23.6%)	1		
***Birth weight***					
*Very low birthweight*	56	13 (28.0%)	1.15 (0.69–1.91)		NS
*Low birthweight*	207	41 (22.7%)	0.93 (0.63–1.37)		
*Normal birthweight*	647	153 (24.4%)	1		
***Length of stay in NNU***					
*24 h or less*	225	40 (17.7%)		1	NS
*25 h to 7 days*	391	90 (24.1%)		1.37 (0.93–2.01)*	
*8 to 28 days*	165	36 (20.3%)		1.15 (0.72–1.82)*	
*More than 28 days*	85	22 (32.4%)		1.84 (1.12–3.01)*	
***Mode of birth***					
*Vaginal*	365	79 (21.9%)		1	NS
*Assisted vaginal*	143	33 (25.7%)		1.17 (0.73–1.88)	
*Planned caesarean*	154	39 (24.9%)		1.14 (0.78–1.66)	
*Unplanned caesarean*	268	60 (25.2%)		1.15 (0.82–1.61)	
***Antenatal anxiety***					
*Yes*	194	93 (49.8%)		3.04 (2.34–3.95)*	1.65 (1.19–2.27)
*No*	738	117 (16.4%)		1	1
***Antenatal depression***					
*Yes*	74	41 (54.7%)		2.65 (1.99–3.54)*	NS
*No*	858	169 (20.6%)		1	
***Smoking during pregnancy***					
*Yes*	61	25 (36.5%)		1.81 (1.21–2.72)*	NS
*No*	595	113 (20.2%)		1	
***Household smoking/passive smoking during pregnancy***					
*Yes*	152	36 (24.1%)		1.02 (0.68–1.53)	NS
*No*	758	167 (23.6%)		1	
***Survey year***					
*2018*	485	82 (19.4%)		1	1
*2020*	450	129 (27.8%)		1.44 (1.08–1.90)*	1.17 (0.89–1.53)

n (unweighted)

% (weighted)

*uRR / aRR* unadjusted risk ratio / adjusted risk ratio

^*^Statistically significant (*p* < 0.1) and entered multivariable model building

^**^Mutually adjusted for social support, long-term mental health problem, satisfaction with labour and birth, antenatal anxiety, in addition to survey year

^#^ Entered into regression analysis as a continuous variable, present Total Number (Median, IQR)

NS risk factor not statistically significant (*p* < 0.05) after multivariable model building

### Factors associated with postnatal anxiety six months after giving birth for mothers whose infants were admitted to NNU

Table 4 describes the prevalence of postnatal anxiety for mothers whose infants were admitted to NNU, according to sociodemographic and pregnancy- and birth-related factors, along with unadjusted and adjusted RRs for the association between risk factors and postnatal anxiety. At the univariable stage, the factors significantly associated with postnatal anxiety were maternal age, country of birth, age when leaving education, social support, having a long-term mental health problem, satisfaction with labour and birth, pregnancy planning, antenatal anxiety, antenatal depression, and smoking during pregnancy. In the final multivariable model, having a long-term mental health problem (aRR 2.16; 95% CI 1.45, 3.23) and antenatal anxiety (aRR 3.35; 95% CI 2.17, 5.18) were significantly associated with postnatal anxiety after adjusting for all other factors. Having social support (aRR 0.87; 95% CI 0.80, 0.96) and higher satisfaction with labour and birth (aRR 0.93; 95% CI 0.88, 0.97) were also protective against postnatal anxiety at six months after birth.

**Table 4:** Risk ratios showing association between postnatal anxiety and sociodemographic and pregnancy and birth-related factors

	**Total** ***N* = 935**	**Postnatal Anxiety** **n (%)**	**uRR (95% CI)**	aRR (95% CI)**
**Sociodemographic factors**				
***Age group***				
≤ *24 years*	93	24 (23.4%)	1.33 (0.82–2.17)*	NS
*25–29 years*	213	33 (15.4%)	0.88 (0.55–1.39)*	
*30–34 years*	337	51 (17.6%)	1	
*35* + *years*	292	28 (9.5%)	0.54 (0.34–0.86)*	
***Country of birth***				
*UK*	734	114 (17.9%)	1	NS
*Outside UK*	197	21 (11.8%)	0.66 (0.40–1.07)*	
***Ethnicity***				
*White-British*	780	115 (16.6%)	1	NS
*Other*	128	14 (12.0%)	0.72 (0.40–1.32)	
***IMD quintile***				
*(Least advantaged) 1*	164	28 (18.2%)	1.73 (0.99–3.03)	NS
*2*	192	37 (19.7%)	1.86 (1.09–3.18)	
*3*	187	25 (14.0%)	1.33 (0.74–2.37)	
*4*	209	26 (13.4%)	1.27 (0.72–2.25)	
*(Most advantaged) 5*	183	20 (10.6%)	1	
***Age when leaving education***				
*16 years or less*	111	25 (22.5%)	1.76 (1.11–2.77)*	NS
*17–18 years*	247	41 (18.1%)	1.42 (0.94–2.15)*	
*19 years or over*	564	66 (12.8%)	1	
***Living with partner***				
*Yes*	836	117 (15.6%)	1	NS
*No*	99	19 (17.5%)	1.12 (0.67–1.87)	
***Social support ***^***#***^				
	932 (6, 4–7)	136 (5, 4–7)	0.83 (0.76–0.91)*	0.87 (0.80–0.96)
***Long-term mental health problem***				
*Yes*	118	56 (51.0%)	4.79 (3.50–6.57)*	2.16 (1.45–3.23)
*No*	810	79 (10.6%)	1	1
**Pregnancy and birth related factors**				
***Parity***				
*Primiparous*	595	83 (15.7%)	1	NS
*Multiparous*	324	52 (16.9%)	1.08 (0.75–1.55)	
***Multiplicity***				
*Singleton*	877	131 (16.4%)	1	NS
*Multiple birth*	57	5 (10.1%)	0.61 (0.25–1.52)	
***Pregnancy Planning***				
*Planned*	735	98 (14.5%)	1	NS
*Unplanned*	189	38 (21.0%)	1.45 (0.99–2.12)*	
***Satisfaction with labour and birth ***^***#***^				
	888 (7, 5–9)	126 (5, 3–9)	0.87 (0.81–0.92)*	0.93 (0.88–0.97)
***Gestation at birth***				
*Very pre-term*	62	10 (17.5%)	1.06 (0.56–1.97)	NS
*Pre-term*	233	31 (14.6%)	0.88 (0.57–1.34)	
*Term*	630	94 (16.6%)	1	
***Birth weight***				
*Very low birthweight*	56	8 (14.7%)	0.88 (0.43–1.79)	NS
*Low birthweight*	207	31 (15.9%)	0.95 (0.63–1.45)	
*Normal birthweight*	647	96 (16.7%)	1	
***Length of stay in NNU***				
*24 h or less*	225	27 (13.4%)	1	NS
*25 h to 7 days*	391	61 (18.0%)	1.35 (0.83–2.19)	
*8 to 28 days*	165	22 (12.6%)	0.94 (0.52–1.70)	
*More than 28 days*	85	15 (20.6%)	1.54 (0.80–2.98)	
***Mode of birth***				
*Vaginal*	365	51 (14.4%)	1	NS
*Assisted vaginal*	143	24 (15.0%)	1.04 (0.63–1.73)	
*Planned caesarean*	154	21 (15.8%)	1.10 (0.65–1.86)	
*Unplanned caesarean*	268	40 (19.4%)	1.35 (0.87–2.08)	
***Antenatal anxiety***				
*Yes*	194	74 (42.5%)	4.93 (3.55–6.86)*	3.35 (2.17–5.18)
*No*	738	62 (8.6%)	1	1
***Antenatal depression***				
*Yes*	74	32 (47.9%)	3.73 (2.64–5.25)*	NS
*No*	858	104 (12.9%)	1	
***Smoking during pregnancy***				
*Yes*	61	21 (30.4%)	2.05 (1.28–3.27)*	NS
*No*	595	80 (14.9%)	1	
***Household smoking/passive smoking during pregnancy***				
*Yes*	152	30 (17.8%)	1.14 (0.75–1.74)	NS
*No*	758	102 (15.6%)	1	
***Survey year***				
*2018*	485	72 (17.3%)	1	1
*2020*	450	64 (14.8%)	0.86 (0.60–1.23)	0.65 (0.48–0.89)

n (unweighted)

% (weighted)

*uRR / aRR* unadjusted risk ratio / adjusted risk ratio

^*^Statistically significant (*p* < 0.1)

^**^Mutually adjusted for social support, long-term mental health problem, satisfaction with labour and birth, antenatal anxiety, in addition to survey year

^#^ Entered into regression analysis as a continuous variable, present Total Number (Median, IQR)

NS risk factor not statistically significant (*p* < 0.05) after multivariable model building

### Factors associated with postnatal PTS six months after giving birth for mothers whose infants were admitted to NNU

Table 5 shows the prevalence of postnatal PTS for mothers whose infants were admitted to NNU, according to sociodemographic and pregnancy- and birth-related factors, in addition to unadjusted and adjusted RRs for the association between risk factors and PTS six months after giving birth. At the univariable stage, the factors significantly associated with postnatal PTS were country of birth, ethnicity, social support, having a long-term mental health problem, pregnancy planning, satisfaction with labour and birth, gestational age, length of NNU stay, mode of birth, antenatal anxiety, antenatal depression, and smoking during pregnancy. In the final multivariable model, having a long-term mental health problem (aRR 2.14; 95% CI 1.43, 3.21) and antenatal anxiety (aRR 2.62; 95% CI 1.71, 4.01) remained significantly associated with PTS after mutually adjusting for all significant risk factors. Having social support (aRR 0.86; 95% CI 0.78, 0.94) and higher satisfaction with labour and birth (aRR 0.89; 95% CI 0.85, 0.94) were also protective against postnatal PTS at six months after birth.

**Table 5:** Risk ratios showing association between postnatal PTS and sociodemographic and pregnancy and birth-related factors

	**Total** ***N* = 935**	**Postnatal** **PTS** **n (%)**	**uRR (95% CI)**		aRR (95% CI)**
**Sociodemographic factors**					
***Age group***					
≤ *24 years*	93	18 (16.4%)	1.25 (0.71–2.22)		NS
*25–29 years*	213	33 (16.1%)	1.23 (0.76–2.00)		
*30–34 years*	337	40 (13.0%)	1		
*35* + *years*	292	43 (14.0%)	1.07 (0.69–1.67)		
***Country of birth***					
*UK*	734	118 (17.3%)	1		NS
*Outside UK*	197	16 (9.1%)	0.52 (0.30–0.92)*		
***Ethnicity***					
*White-British*	780	122 (16.4%)	1		NS
*Other*	128	8 (7.0%)	0.42 (0.19–0.94)*		
***IMD quintile***					
*(Least advantaged) 1*	164	33 (18.4%)	1.45 (0.86–2.46)		NS
*2*	192	25 (12.7%)	1.00 (0.56–1.80)		
*3*	187	26 (13.2%)	1.04 (0.59–1.82)		
*4*	209	27 (13.8%)	1.09 (0.63–1.87)		
*(Most advantaged) 5*	183	23 (12.7%)	1		
***Age when leaving education***					
*16 years or less*	111	22 (18.8%)	1.41 (0.87–2.28)		NS
*17–18 years*	247	34 (15.8%)	1.19 (0.77–1.82)		
*19 years or over*	564	77 (13.3%)	1		
***Living with partner***					
*Yes*	836	116 (14.6%)	1		NS
*No*	99	18 (14.6%)	1.00 (0.59–1.70)		
***Social support ***^***#***^					
	932 6 (4–7)	132 (5,3–7)	0.81 (0.74–0.89)*		0.86 (0.78–0.94)
***Long-term mental health problem***					
*Yes*	118	52 (46.5%)	4.74 (3.43–6.54)*		2.14 (1.43–3.21)
*No*	810	82 (9.8%)	1		1
**Pregnancy and birth related factors**					
***Parity***					
*Primiparous*	595	79 (14.3%)	1		NS
*Multiparous*	324	55 (15.8%)	1.10 (0.77–1.59)		
***Multiplicity***					
*Singleton*	877	123 (14.3%)	1		NS
*Multiple birth*	57	11 (20.3%)	1.41 (0.77–2.58)		
***Pregnancy Planning***					
*Planned pregnancy*	735	97 (13.3%)	1		NS
*Unplanned pregnancy*	189	37 (19.1%)	1.44 (0.97–2.13)*		
***Satisfaction with labour and birth ***^***#***^					
	888 (7, 5–9)	129 (5, 3–8)	0.84 (0.79–0.89)*		0.89 (0.85–0.94)
***Gestation at birth***					
*Very pre-term*	62	16 (27.7%)	2.06 (1.25–3.40)*		NS
*Pre-term*	233	35 (15.3%)	1.14 (0.75–1.73)*		
*Term*	630	82 (13.4%)	1		
***Birth weight***					
*Very low birthweight*	56	14 (23.3%)	1.60 (0.92–2.77)		NS
*Low birthweight*	207	26 (13.9%)	0.95 (0.61–1.49)		
*Normal birthweight*	647	93 (14.6%)	1		
***Length of stay in NNU***					
*24 h or less*	225	19 (9.7%)	1		NS
*25 h to 7 days*	391	59 (14.9%)	1.53 (0.88–2.68)*		
*8 to 28 days*	165	28 (16.3%)	1.68 (0.91–3.10)*		
*More than 28 days*	85	20 (26.1%)	2.69 (1.41–5.15)*		
***Mode of birth***					
*Vaginal*	365	51 (14.4%)	1		NS
*Assisted vaginal birth*	143	17 (10.3%)	0.71 (0.40–1.27)*		
*Planned caesarean*	154	20 (11.1%)	0.77 (0.44–1.34)*		
*Unplanned caesarean*	268	45 (19.5%)	1.36 (0.89–2.06)*		
***Antenatal anxiety***					
*Yes*	194	69 (38.9%)	4.94 (3.54–6.88)*		2.62 (1.71–4.01)
*No*	738	65 (7.9%)	1	1	
***Antenatal depression***					
*Yes*	74	34 (48.4%)	4.28 (3.04–6.05)*		NS
*No*	858	100 (11.3%)	1		
***Smoking during pregnancy***					
*Yes*	61	21 (29.3%)	2.19 (1.36–3.54)*		NS
*No*	595	77 (13.4%)	1		
***Household smoking/passive smoking during pregnancy***					
*Yes*	152	25 (13.6%)	0.90 (0.57–1.41)		NS
*No*	758	105 (15.2%)	1		
***Survey year***					
*2018*	485	58 (12.9%)	1		1
*2020*	450	76 (16.3%)	1.27 (0.88–1.82)		0.90 (0.65–1.24)

n (unweighted)

% (weighted)

^*^Statistically significant (*p* < 0.1)

^**^Mutually adjusted for social support, long-term mental health problem, satisfaction with labour and birth, antenatal anxiety, in addition to survey year

^#^ Entered into regression analysis as a continuous variable, present Total Number (Median, IQR)

NS risk factor not statistically significant (*p* < 0.05) after multivariable model building

*uRR / aRR* unadjusted risk ratio / adjusted risk ratio

As long-term mental health problems and antenatal anxiety were the strongest risk factors for all three outcomes, the degree of overlap between these factors was calculated. Of the 925 women who had their baby admitted to neonatal care and who indicated whether or not they had long-term mental health problems and antenatal anxiety, 7.9% reported a long-term mental health problem and antenatal anxiety, 4.9% reported a long-term mental health problem but no antenatal anxiety, 12.9% reported antenatal anxiety but no long-term mental health problem, and 74.4% reported no long-term mental health problem or antenatal anxiety (Table S1 in Supplementary File 1).

### Factors associated with having one, two, or three postnatal mental health problems for mothers whose infants were admitted to NNU six months after giving birth

Tables S2 and S3 (see Supplementary Files 2 and 3) summarise the unadjusted and adjusted odds ratios for the associations between sociodemographic and pregnancy- and birth-related factors and one postnatal mental health problem (depression, anxiety, or PTS) and two or three postnatal mental health problems (comorbid depression, anxiety, and/or PTS). The multinomial analyses indicated that, compared to women who reported no long-term mental health problem, those who had a long-term mental health problem had an increased odds of having one postnatal mental health problem (depression, anxiety, or PTS) (aOR 3.06; 95% CI: 1.53, 6.15) and greater odds of having two or three postnatal mental health problems (aOR 8.37; 95% CI: 4.35, 16.12) six months after giving birth. Similarly, those women with antenatal anxiety had an increased odds of having one postnatal mental health problem (aOR 3.16; 95% CI: 1.74, 5.73) and greater odds of having two or three postnatal mental health problems (aOR 5.63; 95% CI: 3.19, 9.93) six months after birth, compared with women who did not report antenatal anxiety. Social support was a protective factor and decreased the odds of having one postnatal mental health problem (aOR 0.70; 95% CI: 0.60, 0.81) or two or three postnatal mental health problems (aOR 0.65; 95% CI: 0.56, 0.75) six months after giving birth. Similarly, satisfaction with labour and birth was a protective factor and decreased the odds of having one postnatal mental health problem (aOR 0.88; 95% CI: 0.81, 0.96) or two or three postnatal mental health problems (aOR 0.81; 95% CI: 0.74, 0.88) six months after giving birth.

## Discussion

### Summary of findings

Results from this representative population-based study suggest that approximately one in four mothers whose infants were admitted to NNU (23.7%) experienced postnatal depression six months after birth, while 16.0% and 14.6% experienced anxiety and PTS respectively. A third (31.1%) of mothers whose infants were admitted to NNU experienced at least one mental health problem, with 8.2% experiencing two mental health problems and 7.5% experiencing all three mental health problems six months after birth. Study findings suggest that the strongest risk factors associated with postnatal depression, anxiety, PTS, and comorbidity of these mental health problems include having a long-term mental health problem and antenatal anxiety. Having a self-reported long-term mental health problem was associated with an approximate two-fold increase in postnatal depression, anxiety, and PTS, and an eight-fold increase in comorbidity of mental health problems. Compared to women who did not report antenatal anxiety, women who reported anxiety experienced an approximate two-fold increase in postnatal depression, along with a three-fold increase in postnatal anxiety and PTS six months after childbirth. Women with antenatal anxiety also experienced six times greater odds of having comorbidity of postnatal mental health problems compared to women who did not report antenatal anxiety. There was a degree of overlap between long-term mental health problems and antenatal anxiety. However, the results of the multivariable analyses for all three outcomes showed that long-term mental health problems and antenatal anxiety are strong and independent risk factors for postnatal depression, anxiety and PTS.

Study findings also suggest that having greater social support and higher satisfaction with labour and birth are both protective against all three postnatal mental health problems and comorbidities. Every unit increase in social support (on a scale from 0 to 6) was associated with a 22% lower risk of postnatal depression, 13% lower risk of postnatal anxiety, 14% lower risk of postnatal PTS, and 35% lower risk of comorbidity of these mental health problems six months after giving birth. Every unit increase in satisfaction with labour and birth (on the BSS-RI scale from 0 to 12) was associated with a 6–11% decrease in risk of postnatal depression, anxiety, and PTS and a 19% decrease in risk of comorbidity of these mental health problems six months after giving birth.

In comparison to current study findings, a recent meta-analysis conducted by Malouf et al. [2] estimated a 26.3% prevalence of anxiety and 24.5% prevalence for PTS in mothers whose infants were admitted to NNU between one month to one year postnatal. These prevalence rates are higher than our current study results, but discrepancies could be explained by differences in study sample, assessment tools, as well as time of assessment. However, all estimates in the review were pooled from a small number of studies with high heterogeneity, where individual prevalence ranged from 6.9% to 42.5% for anxiety, and 5.8% to 58.8% for PTS from one month up to one year after birth [2]. Das et al. [9] found that 15.6% of mothers whose infants were admitted to NNU reported symptoms of comorbid postnatal depression, anxiety, and stress in a sample of 118 women in the USA. Although researchers also employed survey-based methodology, higher prevalence rates of triple-comorbidity in this study compared to current study findings could be explained by the small sample size and timing of survey administration, where mothers were approached and screened prior to their infants being discharged from the NNU.

Having a long-term mental health problem and antenatal anxiety were the strongest risk factors predicting postnatal depression, anxiety, PTS, and comorbid mental health problems in the current study. Results from a meta-analysis demonstrated that history of psychiatric illness is a strong risk factor predicting postnatal depression within the general perinatal population [19]. Current results are also corroborated by other research indicating that prior history of depression and anxiety are important risk factors predicting postnatal depression, anxiety, as well as PTS for mothers of infants admitted to NNU [7, 20]. In contrast, a prospective cohort study conducted by Rogers et al. [21] found that prior history of mental health concerns, especially anxiety and depression, did not predict postnatal depression and anxiety for mothers of preterm infants admitted to the NNU. However, Zaers et al. [22] explained that underreporting prior mental health concerns and the use of self-reported measures instead of clinical interviews could explain this lack of association. Similarly, researchers also found antenatal anxiety to be the most significant risk factor predicting depression, anxiety, and PTS at six months after birth [22]. Despite being a risk factor predicting postnatal depression, anxiety, and PTS in the perinatal population, antenatal depression was not found to be associated with postnatal anxiety, depression, or PTS in the current study. It is hypothesized that assessment of antenatal depression with a single, direct question may have introduced underreporting. As noted by Fellmeth et al., [23] some mothers might be reluctant to disclose or self-identify as depressed when faced with a direct question, due to stigma surrounding perinatal depression. Although antenatal anxiety was also assessed using a single question, underreporting might be less as the term anxiety is more often used in everyday language, thus it may not have the same pathological connotation as “depression.”

Social support and satisfaction with labour and birth were found to be protective against individual and comorbid mental health problems. Lack of social support has been frequently cited in previous research as a significant risk factor predicting postnatal depression and anxiety for mothers whose infants were admitted to NNU, where those who report higher levels of perceived or self-reported social support show fewer symptoms of both postnatal depression and anxiety [24]. Although there are fewer studies examining PTS in mothers of NNU infants compared to postnatal depression or anxiety, studies have concluded that increased social support is associated with decreased PTS, both for mothers of NNU infants and for postnatal mothers in general [25]. Similar to current study results, maternal satisfaction with childbirth has been associated with decreased symptoms of postnatal depression, anxiety, and birth-related PTSD in the general perinatal population [26, 27]. However, there is a lack of research examining birth satisfaction and postnatal mental health outcomes for parents of infants admitted to NNU.

### Strengths and limitations

This study is the first to use a large population-based sample to explore the prevalence and risk factors for postnatal depression, anxiety, PTS, and comorbidity of these mental health problems among mothers of NNU infants, compared with mothers of infants not admitted to NNU from the same population. Previous studies examining mental health outcomes in mothers whose infants were admitted to NNU have included small samples. For example, in a recent meta-analysis [2], the sample sizes of included studies ranged from only 29 to 600 participants. Our study provides a significantly larger sample size, enabling more reliable prevalence estimates. Furthermore, information was available on non-respondents to the survey, which enabled the calculation of survey weights to mitigate the effects of non-response bias.

As this study employed a cross-sectional design, causality between risk factors and mental health outcomes cannot be determined. Participants were asked to recall experiences during pregnancy, which introduces possible recall bias given that surveys were distributed six months postnatally. Women experiencing poor mental health at the time of survey administration may have been more likely to report negative experiences and poorer antenatal mental health. Women who were still in the hospital following extremely preterm birth, whose babies were still admitted to NNU, and those experiencing severe mental health problems at the time of survey administration may also have been less likely to respond. Further, although the included risk factors were identified by a rigorous literature search, this study is a secondary analysis of NMS data, and some risk factors for postnatal depression, anxiety, and PTS identified by previous studies were not assessed in the NMS. For example, previously cited factors such as life stress, intimate partner violence, and previous miscarriage, were not included in the NMS [5, 6, 10]. Furthermore, we were unable to differentiate between different types of NNU, and it is possible that the level of care provided by the NNU might affect maternal mental health outcomes. Another limitation of the current study is that prevalence estimates were based on self-report measures instead of the recommended gold standard clinical interview [28]. In addition, there is conflicting evidence for the performance of the GAD-2 in perinatal populations [29, 30] and limited evidence for the PC-PTSD-5. However, all of the self-report measures selected are validated tools with established cut-off points, facilitating comparison with other studies. Furthermore, the National Institute for Health and Care Excellence (NICE) in the UK specifically recommends the EPDS and GAD-2 for use during pregnancy and the postnatal period [31].

### Implications for research and practice

Our findings highlight the importance of mental health screening for mothers of infants admitted to NNU and postnatal mothers more broadly. Routine screening of mothers whose infants were admitted to NNU may be an effective way of identifying those women with poor mental health. NICE recommends assessing mental health symptoms of all women during their first visit to the general practitioner during pregnancy and in the early postnatal period. However, this postnatal visit for mothers may be missed if their infant(s) remains within the NNU. Parents of infants admitted to NNU may require repeated mental health assessments over the longer-term [32]. Additionally, mental health screening during pregnancy should also aim to identify risk factors for postnatal mental health problems including low social support and pre-existing or ongoing mental health problems to ensure that those women at risk receive support and adequate follow up.

A recent systematic review outlined the feasibility of a universal screening program for postnatal mood and anxiety disorders among caregivers of infants admitted to NNU using short screening tools [33]. Given the routine use of short screening tools such as the GAD-2 [14] for anxiety or the Whooley [34] questions for depression within clinical settings, future studies could evaluate the feasibility and effectiveness of combining such tools to inform the creation of a standardized, universal mental health screening program for parents of infants admitted to NNU. In addition to mental health screening, supportive and preventive interventions for mothers and caregivers of NNU infants also require more attention [33]. As both social support and long-term mental health problems emerged as significant risk factors for all mental health outcomes within this study, emphasis could be placed on peer support and family-centered programs, as well as interventions targeting mothers with pre-existing mental health conditions.

Prior research on peer support programs within the NNU demonstrated that receiving peer support has been found to decrease parental stress, anxiety, and depression through increasing empowerment, confidence, and adaptive coping [35]. Family-integrated care is also increasingly emphasized within NNUs, where healthcare should be provided in the context of the patient, family, as well as their community [36]. Results from a randomized controlled trial demonstrated that family-integrated care is effective at reducing NNU-related stress and anxiety for mothers [34]. Therefore, peer support or family-integrated mental health support programs could also be incorporated into routine care for mothers of infants admitted to NNU, with emphasis on mothers with identified risk factors.

## Conclusion

Results from this study demonstrate consistently higher prevalence of postnatal depression, anxiety, PTS, and comorbidity of these conditions among mothers whose infants were admitted to NNU, compared to mothers whose infants were not admitted. Mothers with a long-term mental health problem and antenatal anxiety were at higher risk of experiencing postnatal mental health problems. However, women with greater social support and higher satisfaction with labour and birth reported lower risk of postnatal mental health problems. In addition to routine and repeated mental health screening for caregivers within the NNU, women with identified risk factors should be identified early and offered appropriate support and follow-up.

## Supplementary Information

**Additional file 1: Table S1.** Overlap between long-term mental health problems and antenatal anxiety.

**Additional file 2: Table S2.** Unadjusted risk ratios showing association between any one or any two mental health conditions and sociodemographic and pregnancy and birth-related factors.

**Additional file 3: Table S3.** Adjusted risk ratios showing association between any one or any two mental health conditions and sociodemographic and pregnancy and birth-related factors.

## Data Availability

The datasets used and/or analysed during the current study are available from the corresponding author on reasonable request.
