# Supplementary material for: Prevalence and risk factors for postnatal mental health problems in mothers of infants admitted to neonatal care: analysis of two population-based surveys in England
Source: BMC Pregnancy Childbirth. 2023 May 22;23:370. doi: 10.1186/s12884-023-05684-5 (PMC10201804; doi:10.1186/s12884-023-05684-5)
Supplement: Supplementary file 1 — Additional file 1: Table S1. Overlap between long-term mental health problems and antenatal anxiety. [file 12884_2023_5684_MOESM1_ESM.docx]

**Supplementary File 1**

**Table S1: Overlap between long-term mental health problems and antenatal anxiety**

| **Total N=925** |  | **Antenatal anxiety** | |
| --- | --- | --- | --- |
|  |  | **Yes** | **No** |
| **Long-term mental health problem** | **Yes** | 73 (7.9%) | 45 (4.9%) |
|  | **No** | 119 (12.9%) | 688 (74.4%) |
