# Supplementary material for: Prevalence and risk factors for postnatal mental health problems in mothers of infants admitted to neonatal care: analysis of two population-based surveys in England
Source: BMC Pregnancy Childbirth. 2023 May 22;23:370. doi: 10.1186/s12884-023-05684-5 (PMC10201804; doi:10.1186/s12884-023-05684-5)
Supplement: Supplementary file 2 — Additional file 2: Table S2. Unadjusted risk ratios showing association between any one or any two mental health conditions and sociodemographic and pregnancy and birth-related factors. [file 12884_2023_5684_MOESM2_ESM.docx]

**Supplementary File 2**

**Table S2: Unadjusted risk ratios showing association between any one or any two mental health conditions and sociodemographic and pregnancy and birth-related factors**

|  | **Total**  **N=935** | **Any one condition**  **n(%)** | **Two or more conditions**  **n(%)** | **uRR Any one vs no conditions** | **uRR Two or more conditions vs no conditions** |
| --- | --- | --- | --- | --- | --- |
| **Sociodemographic factors** | | | | | |
| ***Age group*** | | | | | |
| *≤24 years* | 93 | 17 (21.7%) | 22 (19.1%) | 2.10 (0.98-4.49) | 1.31 (0.69-2.48) |
| *25-29 years* | 213 | 31 (12.6%) | 33 (16.1%) | 1.01 (0.57-1.78) | 0.92 (0.53-1.59) |
| *30-34 years* | 337 | 40 (12.3%) | 53 (17.4%) | 1 | 1 |
| *35+ years* | 292 | 54 (17.2%) | 32 (11.1%) | 1.38 (0.83-2.28) | 0.63 (0.37-1.06) |
| ***Country of birth*** | | | | | |
| *UK* | 734 | 116 (15.7%) | 119 (17.9%) | 1 | 1 |
| *Outside UK* | 197 | 25 (14.2%) | 20 (11.0%) | 0.80 (0.45-1.43) | 0.55 (0.31-0.96) |
| ***Ethnicity*** | | | | | |
| *White-British* | 780 | 113 (14.9%) | 124 (17.1%) | 1 | 1 |
| *Other* | 128 | 23 (16.7%) | 11 (9.6%) | 1.03 (0.58-1.86) | 0.52 (0.25-1.09) |
| ***IMD quintile*** |  |  |  |  |  |
| *(Least advantaged) 1* | 164 | 28 (16.2%) | 31 (17.9%) | 0.91 (0.49-1.69) | 1.91 (0.99-3.67) |
| *2* | 192 | 28 (15.2%) | 32 (16.8%) | 0.82 (0.41-1.67) | 1.73 (0.90-3.32) |
| *3* | 187 | 26 (12.2%) | 28 (15.2%) | 0.62 (0.34-1.14) | 1.47 (0.76-2.83) |
| *4* | 209 | 28 (14.0%) | 29 (15.7%) | 0.73 (0.41-1.33) | 1.56 (0.82-2.97) |
| *(Most advantaged) 5* | 183 | 32 (19.2%) | 20 (10.1%) | 1 | 1 |
| ***Age when leaving education*** | | | | | |
| *16 years or less* | 111 | 12 (10.8%) | 30 (25.9%) | 0.80 (0.37-1.71)* | 2.23 (1.28-3.87)* |
| *17-18 years* | 247 | 43 (16.1%) | 37 (16.3%) | 1.11 (0.68-1.80)* | 1.31 (0.79-2.18)* |
| *19 years or over* | 564 | 83 (15.3%) | 71 (13.2%) | 1 | 1 |
| ***Living with partner*** | | | | | |
| *Yes* | 836 | 120 (14.3%) | 124 (16.2%) | 1 | 1 |
| *No* | 99 | 22 (19.7%) | 16 (13.9%) | 1.44 (0.73-2.85) | 0.90 (0.47-1.73) |
| ***Social support ^#^*** |  |  |  |  |  |
|  | 932 (6, 4-7) | 142 (5, 3-6) | 138 (5, 3-6) | 0.71 (0.62-0.81)* | 0.68 (0.60-0.78)* |
| ***Long-term mental health problem*** | | | | | |
| *Yes* | 118 | 25 (21.1%) | 61 (53.8%) | 4.42 (2.32-8.42)* | 16.24 (9.24-28.55)* |
| *No* | 810 | 116 (14.4%) | 78 (10.0%) | 1 | 1 |

| **Pregnancy and birth related factors** | | | | | |
| --- | --- | --- | --- | --- | --- |
| ***Parity*** |  |  |  |  | |
| *Primiparous* | 595 | 87 (15.9%) | 83 (14.9%) | 1 | 1 |
| *Multiparous* | 324 | 53 (14.7%) | 56 (17.5%) | 0.94 (0.60-1.49) | 1.19 (0.78-1.83) |
| ***Multiplicity*** |  |  |  |  |  |
| *Singleton* | 877 | 134 (15.5%) | 131 (15.7%) | 1 | 1 |
| *Multiple birth* | 57 | 8 (12.3%) | 9 (16.9%) | 0.77 (0.33-1.82) | 1.04 (0.46-2.34) |
| ***Pregnancy Planning*** | | | | | |
| *Planned* | 735 | 107 (14.4%) | 101 (14.7%) | 1.44 (0.87-2.40) | 1.54 (0.95-2.48) |
| *Unplanned* | 189 | 34 (18.1%) | 39 (19.7%) | 1 | 1 |
| ***Satisfaction with labour and birth ^#^*** | | | | | |
|  | 888 (7, 5-9) | 135 (6, 4-9) | 133 (6, 3-8) | 0.88 (0.82-0.94)* | 0.79 (0.74-0.86)* |
| ***Gestation at birth*** |  |  |  |  |  |
| *Very pre-term* | 62 | 14 (22.5%) | 12 (23.6%) | 2.08 (1.02-4.26) | 1.94 (0.93-4.05) |
| *Pre-term* | 233 | 34 (17.1%) | 33 (14.5%) | 1.24 (0.70-2.21) | 0.94 (0.58-1.55) |
| *Term* | 630 | 92 (14.1%) | 94 (15.8%) | 1 | 1 |
| ***Birth weight*** |  |  |  |  |  |
| *Very low birthweight* | 56 | 14 (22.1%) | 10 (20.9%) | 1.73 (0.83-3.63) | 1.56 (0.70-3.46) |
| *Low birthweight* | 207 | 23 (14.8%) | 30 (15.2%) | 0.94 (0.48-1.84) | 0.92 (0.56-1.52) |
| *Normal birthweight* | 647 | 102 (15.4%) | 99 (16.1%) | 1 | 1 |
| ***Length of stay in NNU*** | | | | | |
| *24 hours or less* | 225 | 27 (12.4%) | 24 (11.3%) | 1 | 1 |
| *25 hours to 7 days* | 391 | 62 (16.0%) | 61 (16.6%) | 1.46 (0.83-2.58) | 1.66 (0.92-3.00) |
| *8 to 28 days* | 165 | 22 (11.6%) | 26 (15.3%) | 0.98 (0.48-1.98) | 1.41 (0.72-2.77) |
| *More than 28 days* | 85 | 17 (18.4%) | 17 (24.6%) | 1.98 (0.91-4.27) | 2.91 (1.31-6.45) |
| ***Mode of birth*** |  |  |  |  |  |
| *Vaginal* | 365 | 51 (13.3%) | 54 (15.1%) | 1 | 1 |
| *Assisted vaginal* | 143 | 23 (18.6%) | 21 (13.3%) | 1.47 (0.67-3.22) | 0.93 (0.50-1.71) |
| *Planned caesarean* | 154 | 26 (14.7%) | 23 (15.6%) | 1.13 (0.62-2.07) | 1.07 (0.58-1.96) |
| *Unplanned caesarean* | 268 | 41 (17.0%) | 42 (18.5%) | 1.41 (0.84-2.39) | 1.36 (0.82-2.28) |
| ***Anxiety during current pregnancy*** | | | | | |
| *Yes* | 194 | 44 (24.1%) | 76 (41.4%) | 4.31 (2.59-7.17)* | 10.90 (6.84-17.37)* |
| *No* | 738 | 97 (12.7%) | 64 (8.6%) | 1 | 1 |
| ***Depression during current pregnancy*** | | | | | |
| *Yes* | 74 | 13 (20.2%) | 36 (49.6%) | 3.31 (1.47-7.47)* | 9.62 (5.13-18.04)* |
| *No* | 858 | 128 (14.7%) | 104 (12.4%) | 1 | 1 |
| ***Smoking during pregnancy*** | | | | | |
| *Yes* | 61 | 4 (6.3%) | 25 (35.1%) | 0.56 (0.17-1.85)* | 3.00 (1.58-5.70)* |
| *No* | 595 | 84 (13.8%) | 78 (14.4%) | 1 | 1 |
| ***Household smoking/passive smoking during pregnancy*** | | | | | |
| *Yes* | 152 | 16 (13.5%) | 31 (17.6%) | 0.84 (0.39-1.81) | 1.15 (0.69-1.90) |
| *No* | 758 | 122 (16.0%) | 104 (15.3%) | 1 | 1 |
| ***Survey year*** | | | | | |
| *2018* | 485 | 56 (11.0%) | 65 (15.8%) | 1 | 1 |
| *2020* | 450 | 86 (19.2%) | 75 (15.7%) | 1.96 (1.26-3.06)* | 1.12 (0.73-1.70) |

n (unweighted)

% (weighted)

*Statistically significant (p<0.1)

# Entered into regression analysis as a continuous variable, present Total Number (Median, IQR)
