# Supplementary material for: Prevalence and risk factors for postnatal mental health problems in mothers of infants admitted to neonatal care: analysis of two population-based surveys in England
Source: BMC Pregnancy Childbirth. 2023 May 22;23:370. doi: 10.1186/s12884-023-05684-5 (PMC10201804; doi:10.1186/s12884-023-05684-5)
Supplement: Supplementary file 3 — Additional file 3: Table S3. Adjusted risk ratios showing association between any one or any two mental health conditions and sociodemographic and pregnancy and birth-related factors. [file 12884_2023_5684_MOESM3_ESM.docx]

**Supplementary File 3**

**Table S3: Adjusted risk ratios showing association between any one or any two mental health conditions and sociodemographic and pregnancy and birth-related factors**

|  | **Total**  **N=935** | **Any one condition**  **n(%)** | **Two or more conditions**  **n(%)** | **aOR Any one vs no conditions** | **aOR Two or more conditions vs no conditions** |
| --- | --- | --- | --- | --- | --- |
| **Sociodemographic factors** | | | | | |
| ***Age group*** | | | | | |
| *≤24 years* | 93 | 17 (21.7%) | 22 (19.1%) | NS | NS |
| *25-29 years* | 213 | 31 (12.6%) | 33 (16.1%) |  |  |
| *30-34 years* | 337 | 40 (12.3%) | 53 (17.4%) |  |  |
| *35+ years* | 292 | 54 (17.2%) | 32 (11.1%) |  |  |
| ***Country of birth*** | | | | | |
| *UK* | 734 | 116 (15.7%) | 119 (17.9%) | NS | NS |
| *Outside UK* | 197 | 25 (14.2%) | 20 (11.0%) |  |  |
| ***Ethnicity*** | | | | | |
| *White-British* | 780 | 113 (14.9%) | 124 (17.1%) | NS | NS |
| *Other* | 128 | 23 (16.7%) | 11 (9.6%) |  |  |
| ***IMD quintile*** |  |  |  |  |  |
| *(Least advantaged) 1* | 164 | 28 (16.2%) | 31 (17.9%) |  |  |
| *2* | 192 | 28 (15.2%) | 32 (16.8%) | NS | NS |
| *3* | 187 | 26 (12.2%) | 28 (15.2%) |  |  |
| *4* | 209 | 28 (14.0%) | 29 (15.7%) |  |  |
| *(Most advantaged) 5* | 183 | 32 (19.2%) | 20 (10.1%) |  |  |
| ***Age when leaving education*** | | | | | |
| *16 years or less* | 111 | 12 (10.8%) | 30 (25.9%) |  |  |
| *17-18 years* | 247 | 43 (16.1%) | 37 (16.3%) | NS | NS |
| *19 years or over* | 564 | 83 (15.3%) | 71 (13.2%) |  |  |
| ***Living with partner*** | | | | | |
| *Yes* | 836 | 120 (14.3%) | 124 (16.2%) | NS | NS |
| *No* | 99 | 22 (19.7%) | 16 (13.9%) |  |  |
| ***Social support ^#^*** |  |  |  |  |  |
|  | 932 (6, 4-7) | 142 (5, 3-6) | 138 (5, 3-6) | 0.70 (0.60-0.81)* | 0.65 (0.56-0.75)* |
| ***Long-term mental health problem*** | | | | | |
| *Yes* | 118 | 25 (21.1%) | 61 (53.8%) | 3.06 (1.53-6.15)* | 8.37 (4.35-16.12)* |
| *No* | 810 | 116 (14.4%) | 78 (10.0%) | 1 | 1 |

| **Pregnancy and birth related factors** | | | | | |
| --- | --- | --- | --- | --- | --- |
| ***Parity*** |  |  |  |  | |
| *Primiparous* | 595 | 87 (15.9%) | 83 (14.9%) | NS | NS |
| *Multiparous* | 324 | 53 (14.7%) | 56 (17.5%) |  |  |
| ***Multiplicity*** |  |  |  |  |  |
| *Singleton* | 877 | 134 (15.5%) | 131 (15.7%) | NS | NS |
| *Multiple birth* | 57 | 8 (12.3%) | 9 (16.9%) |  |  |
| ***Pregnancy Planning*** | | | | | |
| *Planned* | 735 | 107 (14.4%) | 101 (14.7%) | NS | NS |
| *Unplanned* | 189 | 34 (18.1%) | 39 (19.7%) |  |  |
| ***Satisfaction with labour and birth ^#^*** | | | | | |
|  | 888 (7, 5-9) | 135 (6, 4-9) | 133 (6, 3-8) | 0.88 (0.81-0.96)* | 0.81 (0.74-0.88)* |
| ***Gestation at birth*** |  |  |  |  |  |
| *Very pre-term* | 62 | 14 (22.5%) | 12 (23.6%) |  |  |
| *Pre-term* | 233 | 34 (17.1%) | 33 (14.5%) | NS | NS |
| *Term* | 630 | 92 (14.1%) | 94 (15.8%) |  |  |
| ***Birth weight*** |  |  |  |  |  |
| *Very low birthweight* | 56 | 14 (22.1%) | 10 (20.9%) | NS | NS |
| *Low birthweight* | 207 | 23 (14.8%) | 30 (15.2%) |  |  |
| *Normal birthweight* | 647 | 102 (15.4%) | 99 (16.1%) |  |  |
| ***Length of stay in NNU*** | | | | | |
| *24 hours or less* | 225 | 27 (12.4%) | 24 (11.3%) |  |  |
| *25 hours to 7 days* | 391 | 62 (16.0%) | 61 (16.6%) | NS | NS |
| *8 to 28 days* | 165 | 22 (11.6%) | 26 (15.3%) |  |  |
| *More than 28 days* | 85 | 17 (18.4%) | 17 (24.6%) |  |  |
| ***Mode of birth*** |  |  |  |  |  |
| *Vaginal* | 365 | 51 (13.3%) | 54 (15.1%) |  |  |
| *Assisted vaginal* | 143 | 23 (18.6%) | 21 (13.3%) | NS | NS |
| *Planned caesarean* | 154 | 26 (14.7%) | 23 (15.6%) |  |  |
| *Unplanned caesarean* | 268 | 41 (17.0%) | 42 (18.5%) |  |  |
| ***Anxiety during current pregnancy*** | | | | | |
| *Yes* | 194 | 44 (24.1%) | 76 (41.4%) | 3.16 (1.74-5.73)* | 5.63 (3.19-9.93)* |
| *No* | 738 | 97 (12.7%) | 64 (8.6%) | 1 | 1 |
| ***Depression during current pregnancy*** | | | | | |
| *Yes* | 74 | 13 (20.2%) | 36 (49.6%) | NS | NS |
| *No* | 858 | 128 (14.7%) | 104 (12.4%) |  |  |
| ***Smoking during pregnancy*** | | | | | |
| *Yes* | 61 | 4 (6.3%) | 25 (35.1%) | NS | NS |
| *No* | 595 | 84 (13.8%) | 78 (14.4%) |  |  |
| ***Household smoking/passive smoking during pregnancy*** | | | | | |
| *Yes* | 152 | 16 (13.5%) | 31 (17.6%) | NS | NS |
| *No* | 758 | 122 (16.0%) | 104 (15.3%) |  |  |
| ***Survey year*** | | | | | |
| *2018* | 485 | 56 (11.0%) | 65 (15.8%) | 1 | 1 |
| *2020* | 450 | 86 (19.2%) | 75 (15.7%) | 1.77 (1.08-2.92)* | 0.72 (0.44-1.20) |

n (unweighted)

% (weighted)

*Statistically significant (p<0.05)

# Entered into regression analysis as a continuous variable, present Total Number (Median, IQR)

NS risk factor not statistically significant (p<0.05) after multivariable model building
